# Applied Stress-Assisted Growth of Single Crystal γ-Fe_2_O_3_ Nanowires

**DOI:** 10.3390/nano8121037

**Published:** 2018-12-12

**Authors:** Lan Lu, Ligen Hong, Yi Chu, Huayong Pan, Shaoyun Huang, Yingjie Xing, Hongqi Xu

**Affiliations:** Bejing Key Laboratory of Quantum Devices, Key Laboratory for the Physics and Chemistry of Nanodevices, and Department of Electronics, Peking University, Beijing 100871, China; lulantx@163.com (L.L.); honglg33@pku.edu.cn (L.H.); 1601214331@pku.edu.cn (Y.C.); hypan@pku.edu.cn (H.P.); syhuang@pku.edu.cn (S.H.)

**Keywords:** nanowires, synthesis, novel phenomena, maghemite, growth model

## Abstract

It is difficult to obtain γ-Fe_2_O_3_ nanostructures by heating iron substrate in ambient conditions because γ-Fe_2_O_3_ is less thermodynamically stable than α-Fe_2_O_3_. In this work, we synthesize γ-Fe_2_O_3_ nanowires by heating iron particles under an external force. The stacking style of iron and oxygen ions under a strong shearing stress tends to adopt the γ-Fe_2_O_3_ structure regardless of the thermodynamic restriction. These γ-Fe_2_O_3_ nanowires exhibit a clear ferromagnetic property. Transmission electron microscopy (TEM) and X-ray diffraction (XRD) measurements confirm that γ-phase structure appears only under the applied external force during the heating period. A window of the magnitude of the external force is found to help the nanowire growth on iron particles. The growth mechanism of γ-Fe_2_O_3_ nanowires other than α-Fe_2_O_3_ under the external force is discussed and an applied stress-assisted growth model is proposed. This work presents an easy approach to produce ferromagnetic iron oxide nanowires on a large scale.

## 1. Introduction

Iron oxide nanowires have attracted intensive interest due to their application in gas sensors, catalysts, Li-ion battery, and ferromagnetic contrast agents [1]. Iron oxide nanowires require cheap, simple and controllable synthesis approaches. Besides the solution method [2,3], direct oxidation of iron substrate at high temperature is another effective and simple method to prepare iron oxide nanowires. Dense Fe_2_O_3_ nanowires and nanobelts are formed on the iron substrate by simple heating treatment in an oxygen-rich atmosphere [4,5,6,7,8]. The study on Fe_2_O_3_ nanowire growth reveals a process determined by the oxidation process of the iron substrate surface.

Iron oxidation has been studied for a long time and is summarized in some classic literature [9]. A Fe-O phase diagram shows that Fe_2_O_3_(top)/Fe_3_O_4_/FeO(bottom) triple-layers are formed on the surface of iron foil higher than 570 °C, whereas Fe_2_O_3_(top)/Fe_3_O_4_(bottom) bilayers are generated at lower than 570 °C. In both cases, the appearance of a compact Fe_2_O_3_ surface layer is independent of the heating temperature. There are two common structures of Fe_2_O_3_, α-Fe_2_O_3_ (hematite) and γ-Fe_2_O_3_ (maghemite). Hematite is the most thermodynamically stable iron oxide under ambient conditions, and γ-Fe_2_O_3_ is a metastable polymorph with a ferromagnetic property. The transformation temperature of γ-Fe_2_O_3_ to α-Fe_2_O_3_ is ~400 °C for bulk material [9]. The thermodynamic stability also influences the phase of nanostructured iron oxides. α-Fe_2_O_3_ nanowires are the common products of heating the iron substrate regardless of heating temperature (255–1000 °C) [4,5,6,7,8]. To date, there has been no report on the synthesis of γ-Fe_2_O_3_ nanowires by heating the iron substrate to the best of our knowledge. The formation of γ-Fe_2_O_3_ nanowires without solution media can only be achieved by flame vapor deposition, in which γ-Fe_2_O_3_ nanowires are found on Fe or Si substrate closing to a directly burned iron mesh [10]. 

A vapor-solid mechanism is proposed to explain the synthesis of iron oxide nanowires at high temperature [4], whereas for lower heating temperature, e.g., ~400 °C, the driving force for nanowire growth is concluded as the compressive stresses induced by the volume variation accompanying the Fe_2_O_3_/Fe_3_O_4_ interface reaction during the surface oxidation of the iron substrate [7,8]. In the latter case, the growth rate of iron oxide nanowire is decided by the surface diffusion of iron cations, which are supplied by Fe cation diffusion through the outward grain boundary in the α-Fe_2_O_3_ layer. Therefore, iron ion diffusion and a grain boundary are two critical conditions for α-Fe_2_O_3_ nanowire growth at lower heating temperature. 

In the framework of the compressive stress-driven model, the effect of the heating temperature on iron ion diffusion is much less than that on the formation of the grain boundary [7,8]. This characteristic means that in order to initiate Fe_2_O_3_ nanowire growth, the oxidation process must produce enough internal stresses to generate sufficient grain boundaries in the α-Fe_2_O_3_ layer at the heating temperature. Since the lowest record of the growth temperature of α-Fe_2_O_3_ nanowire is 255 °C in literature [5], a compact Fe_2_O_3_ layer with negligible grain boundaries is supposed to exist on the iron substrate surface below 255 °C, resulting in the negligible iron ion diffusion to the substrate surface. The lack of γ-Fe_2_O_3_ nanowire by means of heating iron substrate in literature can also be explained by the same mechanism. For the sake of the stable existence of γ-Fe_2_O_3_, a high heating temperature must be avoid. Fewer grain boundaries can be created in the Fe_2_O_3_ layer under this heating condition. There seems to be an intrinsic impediment to the growth of γ-Fe_2_O_3_ nanowires based on the simple heating treatment.

Here, we apply an external force on iron particles during the heating period. This external force produces uneven distribution of applied stresses in the iron particle. Therefore, at some points on the surface of the iron particle, more grain boundaries may be generated in the Fe_2_O_3_ layer by the total stresses, even if the internal compressive stresses themselves are not large enough. Single crystalline γ-Fe_2_O_3_ nanowires are formed directly on iron substrate for the first time by this applied-stress assisted growth method. The growth of γ-Fe_2_O_3_ nanowires in this work is much simpler than previous methodologies [2,3,10].

## 2. Materials and Methods

Iron particles (purity 99.999%, CNM tech., Beijing, China) with the size of several microns are used as both the source and the substrate for nanowire growth. The particles are dispersed on Si/SiO_2_ plate and rinsed in diluted hydrochloric acid. Some Si/SiO_2_ plates with iron particles are placed directly in the center of a tube furnace (Shenjia Co., Luoyang, China). Other Si/SiO_2_ plates with iron particles are covered by another clean Si/SiO_2_ plate and then are sandwiched between two pieces of glass. A clamp is used to clamp the glass to construct a sandwich configuration for heating. In all experiments to find the optimal heating temperature and heating time, a fixed external force is applied on the iron particles. More experiments are carried out to compare the effect of the magnitude of the external force on nanowire growth. The sandwiched samples are heated on a hot plate (Donghangkeyi Co., EH-45, Beijing, China). An accurate pressure is produced by placing a standard weight on top of the sandwiched samples. The heating time is in the range of 6–12 h. We find that longer heating time does not increase the quantity of nanowires significantly. All heating experiments are conducted in air.

The morphology of the samples is observed by scanning electron microscope (SEM, FEI Quanta 600, Hillsboro, OR, USA). Highly magnified morphology, crystal structure and composition are analyzed by a high-resolution transmission electron microscope (HRTEM, FEI Tecnai F30, Hillsboro, OR, USA) equipped energy dispersive X-ray spectroscopy (EDX). The structure of nanowires is measured by an X-ray diffraction (XRD) powder diffractometer (Panalytical X-Pert3 Powder, Almelo, Netherlands). The magnetic property of the nanowires is characterized at 300 K by a superconducting quantum interference device magnetometer (SQUID-VSM, Quantum Design, San Diego, CA, USA). In order to eliminate the influence from the magnetic iron particles, the nanowires are broken from the iron particles in ethanol by strong ultrasonic treatment and are filtered through a filter with hole size of 0.45 μm. Several droplets of ethanol solution are deposited on a clean Si/SiO_2_ plate and then dried naturally for magnetic measurement. Because the mass of the nanowires purified in this way is very small and hard to weigh accurately, we do not use the mass to normalize the measured magnetization in this work.

## 3. Results and Discussion

By contrast with the flat iron film or foil in previous literature [6,7,8,11,12,13], we choose iron particles as the source and substrate to investigate the effect of the external force on nanowire growth in this work. Because of its round sphere shape, the contact area of a single iron particle to another particle or Si/SiO_2_ plate is very small, resulting in a much larger pressure on the round particle than on a flat foil under the same force. Also due to the round sphere shape, the extra stresses produced by the large pressure distribute unevenly in the iron particle. We call such an extra stress the applied stress and propose that the iron particle as a suitable object to observe the effect of the applied stress on nanowire growth. We note that a large density of iron oxide nanowires is not pursued in some experiments at lower growth temperature.

**Figure 1:**
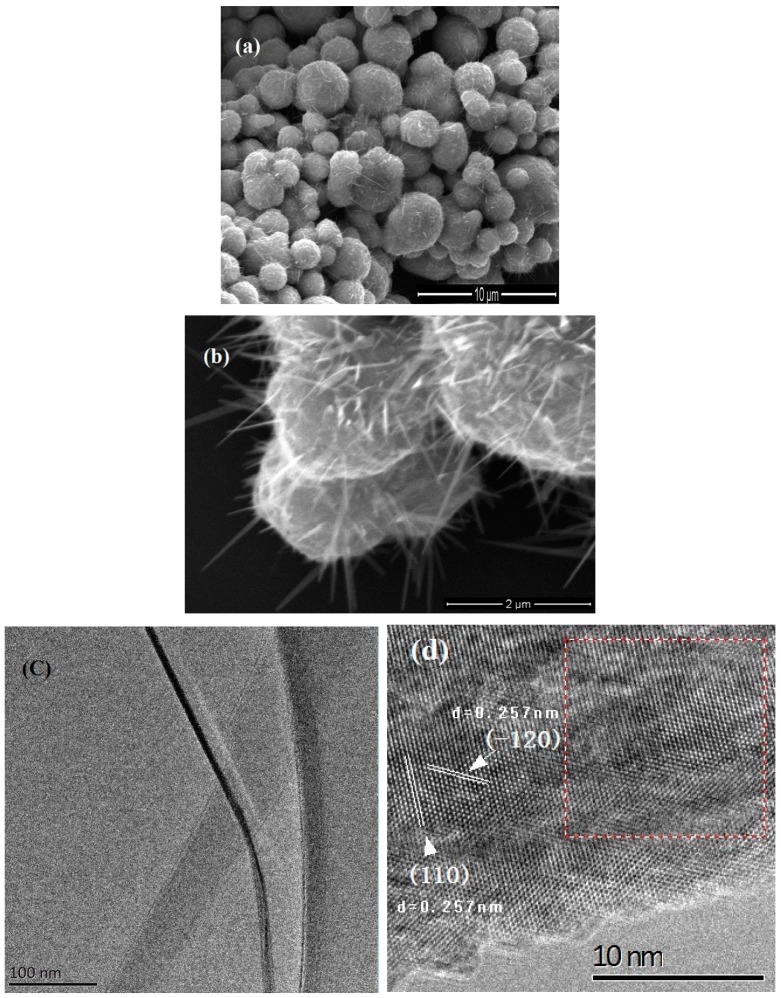

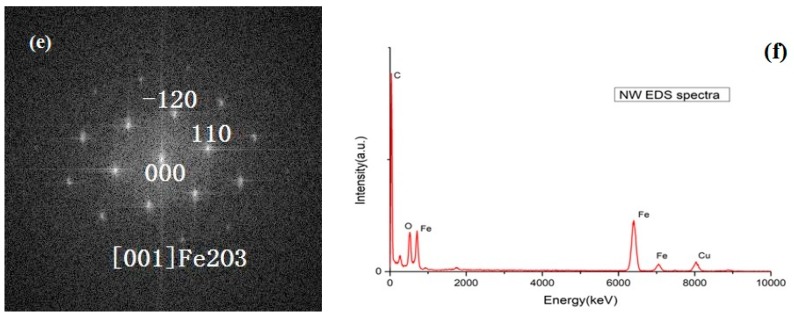
Nanowires grown on iron particles at 300 °C without the external force. (**a**) Low and (**b**) high magnification scanning electron microscope (SEM) image; (**c**) transmission electron microscope (TEM) and (**d**) high-resolution TEM (HRTEM) image of a single α-Fe_2_O_3_ nanowire; (**e**) FFT pattern of the area of red rectangle in (**d**); (**f**) energy dispersive X-ray spectroscopy (EDX) spectrum of a single nanowire.

The shortest time for nanowire growth at 300 °C is 4 h without the external force. We find the length of nanowires enlarges obviously within a 12 h heating period. Figure 1a shows the morphology of nanowires grown at 300 °C after 12 h heating. No force is applied to this sample. Many nanowires protrude from each particle. The nanowires have the diameter in the range of 40–100 nm and the length of several microns. A tapered shape is observed in all nanowires, which is a typical signature of surface diffusion controlled nanowire growth [14,15,16]. A magnified image of the nanowires is shown in Figure 1b. We find the density of nanowires decreases significantly when we reduce the heating temperature to 280 °C. We note this temperature is higher than the lowest growth temperature (255 °C) in Ref. 5. Figure 1c shows a transmission electron microscope (TEM) image of a single nanowire. Figure 1d shows a high magnification TEM image of this nanowire. (110) and (−120) planes with d-spacing of 0.257 nm are observed in the nanowire. Figure 1e shows the FFT pattern of red rectangle area in Figure 1d, which corresponds to α-phase of Fe_2_O_3_. Figure 1f shows the EDX spectrum of the nanowire. Fe and O are detected from the nanowire. Cu and C come from the copper grid. Above result reveals a lot of α-Fe_2_O_3_ nanowires are grown on iron particles after heating at 300 °C. Based on the compressive stress driven model, we think that sufficient grain boundaries are generated in the Fe_2_O_3_ layer by the internal stresses at this temperature (300 °C), and then Fe ions diffuse through these grain boundaries and react with oxygen to form single crystalline α-Fe_2_O_3_ nanowires on iron particle surface.

**Figure 2:**
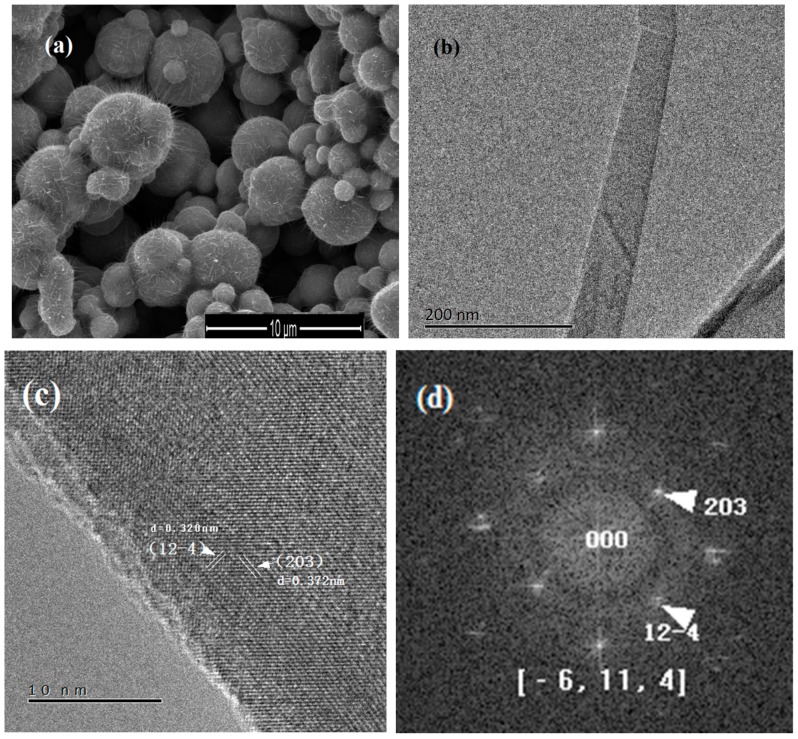

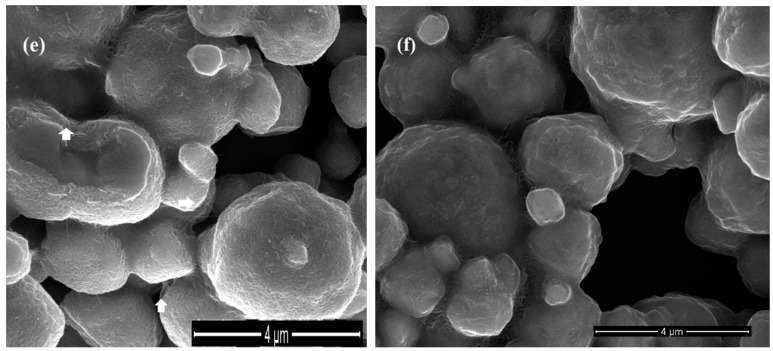
Nanowires grown on iron particles at 300 °C under the external force. (**a**) SEM image; (**b**) TEM and (**c**) HRTEM image of a single γ-Fe_2_O_3_ nanowire; (**d**) FFT pattern of the nanowire in (**d**). SEM image of nanowires grown at 230 °C (**e**) with and (f) without the external force. White arrows in (**e**) indicate the nanowires on the iron particle.

Figure 2a shows a SEM image of the nanowires grown on pressed iron particles after heating at 300 °C for 12 h. The density, diameter, and length of the nanowires in Figure 2a look similar to that in Figure 1a. Tapered morphology also appears in these nanowires. Figure 2b shows a TEM image of a nanowire. Figure 2c shows a high magnification TEM image of this single nanowire; (12-4) and (203) planes are observed in the nanowire. Figure 2d shows the FFT pattern of this nanowire. Both the clear lattices in Figure 2c and the spots in Figure 2d reveal single crystalline γ-Fe_2_O_3_ structure. The above result show the structure of nanowires grown under the external force is γ-Fe_2_O_3_. A benefit of this method is the lower temperature required for nanowire growth. We find the heating temperature for nanowire growth can be decreased to 230 °C in the clamped sandwich configuration. One sample is particularly clamped to show the effect of the external force at lower heating temperature. After covering the whole surface of Si/SiO_2_ plate with iron particles, only half of the plate surface is clamped, and the other half of the plate exposed to air freely. The morphology of particles heated at 230 °C with and without the external force is shown in Figure 2e,f, respectively. White arrows in Figure 2e indicate the location of nanowires; 230 °C is the lowest growth temperature of iron oxide nanowire grown on iron substrate to the best of our knowledge. Heating experiments at 230 °C with and without the external force have been repeated many times. No nanowire is observed on the surface of the iron particles without force at 230 °C (as shown in Figure 2f), whereas on pressed particles, sparse nanowires are always found. The repeatability of nanowire growth at 230 °C under the external force is 100%. This result reveals that the impediment to nanowire growth at 230 °C is overcome by the external force.

**Figure 3:**
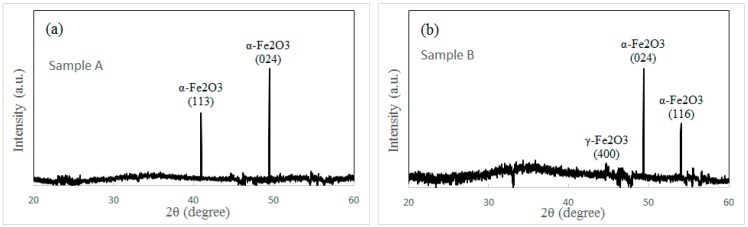

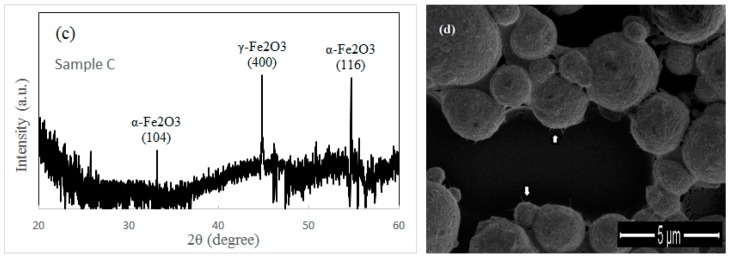
X-ray diffraction (XRD) spectrum of the sample heated (**a**) at 300 °C for 12 h without the external force; (**b**) at 300 °C for 1.5 h under the external force; (**c**) at 300 °C for 12 h under the external force; (**d**) SEM image of sample B. White arrows in (**d**) indicate the nanowires on the iron particle.

More analyses are performed to investigate the structure of iron oxide nanowires grown under different conditions. Three samples are measured by XRD. Sample A is heated at 300 °C for 12 h without the external force (shown in Figure 1a). Sample B is prepared at 300 °C for 1.5 h under the external force. Sample C is heated at 300 °C for 12 h under the external force (shown in Figure 2a). XRD spectra of three samples are shown in Figure 3a–c. Two sharp peaks in sample A (shown in Figure 3a) are indexed to (024) and (113) planes of α-Fe_2_O_3_. This result reflects that only α-Fe_2_O_3_ structure appears in both the nanowires and the surface of the iron particles under the normal oxidization condition. This is in accordance with the conclusion in literature [7,8]. Sample B is prepared in order to answer a question on the effect of the external force. Is there any influence of the external force on the oxidation state of the iron particle? The morphology of sample B is shown in Figure 3d. In contrast to sample A and sample C, only few nanowires are found in sample B after a short heating period (1.5 h). The XRD spectrum of sample B is shown in Figure 3b. We think the oxidized surface of iron particles contributes two strong peaks in Figure 3b. This result confirms that the α-Fe_2_O_3_ layer always appears on the iron particle surface after oxidation whether under the external force or not. A weak peak in Figure 3b is indexed to γ-Fe_2_O_3_, which should come from a small quantity of γ-Fe_2_O_3_ nanowires (shown in Figure 3d). The XRD spectrum of sample C shows two strong peaks of α-Fe_2_O_3_ and one strong peak of γ-Fe_2_O_3_ (shown in Figure 3c). Obviously, the nanowires grown under the external force and the oxidized surface of the iron particles contribute the peak of γ-Fe_2_O_3_ and the other two peaks of α-Fe_2_O_3_, respectively.

**Figure 4:**
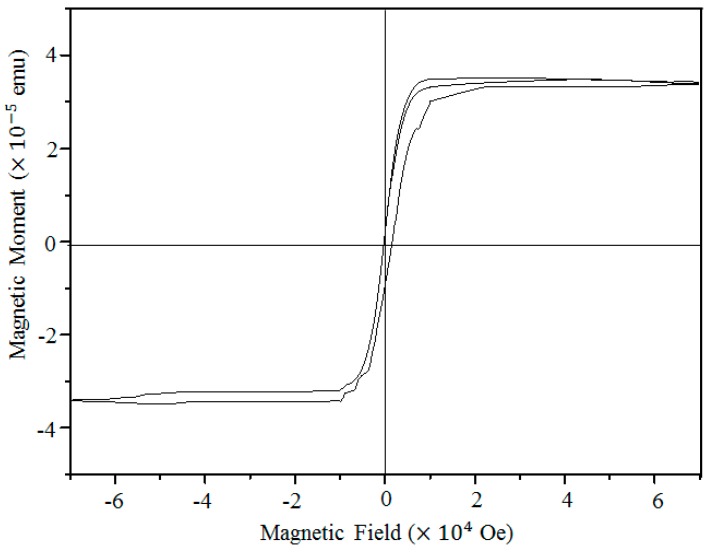
Magnetization vs. field hysteresis loop of γ-Fe_2_O_3_ nanowires measured at 300 K.

We also measure the magnetic property of γ-Fe_2_O_3_ nanowires. Figure 4 shows the magnetization versus field hysteresis loop measured at room temperature. The saturation magnetization (Ms) of a small amount of γ-Fe_2_O_3_ nanowires on Si/SiO_2_ plate is 3.32 × 10^−5^ emu, which is an order of magnitude higher than that of a clean Si/SiO_2_ plate (2.65 × 10^−6^ emu). This ferromagnetic performance clearly confirms the γ-phase of iron oxide nanowires grown under the applied stress.

**Figure 5:**
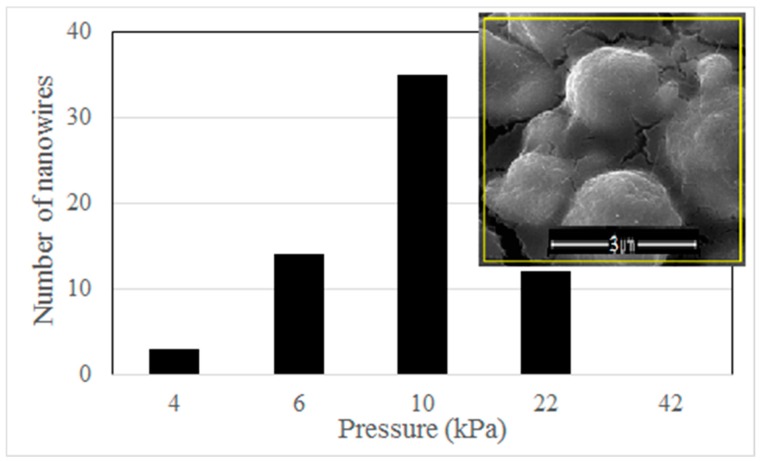
Statistic result of nanowires grown on the hot plate under different external forces. Inset: SEM image of 5 × 5 μm^2^ area (yellow square) under the pressure of 1 × 10^4^ Pa.

The actual magnitude of applied stresses in a single iron particle is difficult to measure. However, we can compare the relative effect of different external forces based on a reasonable assumption. Because the number of iron particles is very large and the stacking style of the iron particles is formed naturally during the drying period in our experiment, we can suppose that the squeezing degree of iron particles in each sample is similar after clamping. Therefore, at the same heating temperature, nanowire density may roughly demonstrate the average effect of the magnitude of the applied stresses. We use a hot plate to heat the iron particles instead of the tube furnace for evaluation of the influence of the force magnitude on nanowire growth. The open space above the hot plate permits a large standard weight pressing down on the sample vertically. Because the area of the Si/SiO_2_ plate can be measured accurately, a precise pressure can be applied on the sample in this way. The heating temperature keeps 300 °C. The density of nanowires decreases significantly in this case, because the heating environment on the hot plate is different from the uniform heating space in the tube furnace. In order to count the number of nanowires accurately, the heating time is shortened to 6 h to further reduce the nanowire amount. We count the number of nanowires in an area of 5 × 5 μm^2^ and find the pressure magnitude has an obvious influence on the density of nanowires. The statistical data of the nanowire number is shown in Figure 5. The optimal pressure is 1 × 10^4^ Pa, under which the largest density of nanowires appears (shown in the inset of Figure 5). We find no nanowire grows under a pressure of 4.2 × 10^4^ Pa. A pressure much larger than this limit even peels the oxide layer after heating treatment. This phenomenon means that a very large external force may be harmful to nanowire growth, although we do not know the exact reason.

**Figure 6:**
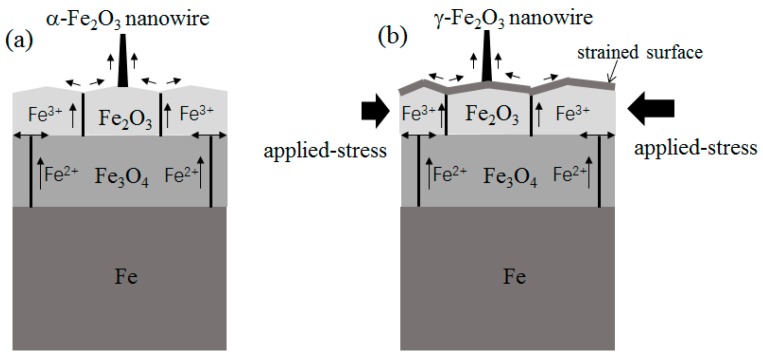
(**a**) Schematic diagram of α-Fe_2_O_3_ nanowire growth without the external force; (**b**) schematic diagram of γ-Fe_2_O_3_ nanowire growth under the external force.

The growth of α-Fe_2_O_3_ nanowires without the external force is in accordance with previous literature and can be understood in the framework of the compressive stress-driven model [7,8]. It has been observed that the formation of grain boundaries occurs quite early during high temperature heating [17]. In the experiment without the external force, sufficient grain boundaries are created as the accumulated effect of the internal stresses at 300 °C. These grain boundaries in the Fe_2_O_3_ layer act as the efficient diffusion route for iron ion diffusion. The growth model of α-Fe_2_O_3_ nanowires is schematically demonstrated in Figure 6a. This mechanism explains the temperature limit (230 °C) for α-Fe_2_O_3_ nanowire growth in both our experiment and Ref. 5. The internal stresses generated in the iron oxide layer are not large enough at 230 °C, resulting in fewer grain boundaries for iron ion diffusion.

A reducing environment has been routinely used to transform γ-Fe_2_O_3_ nanowires from α-Fe_2_O_3_ nanowires [18]. However, the ambient for γ-Fe_2_O_3_ nanowire growth is always the air in our work. This condition means other mechanism than the phase transformation controls γ-Fe_2_O_3_ nanowire growth on top of iron particles. We find the necessary condition for γ-Fe_2_O_3_ nanowire growth is the external force. As mentioned above, we think the principal effect of the external force is to generate applied stresses and induce the grain boundaries quickly and efficiently, because such applied stresses will initiate the generation of grain boundaries in the Fe_2_O_3_ layer from the very beginning of the surface oxidation process. In contrast, a sufficiently long heating time is always needed to accumulate the internal stresses in order to induce sufficient grain boundaries in the case of surface oxidation without the external force. We think that due to the uneven distribution of the applied stresses in a single particle, the nanowire growth should occur near the position bearing the largest total stresses.

There is no report on the direct growth of γ-Fe_2_O_3_ nanowires on iron substrate to date. [1,19] The formation of a less thermodynamically stable γ-phase of Fe_2_O_3_ nanowires by heating must relate to the pressed growth environment. It has been reported that under the stresses applied by high energy ball milling, hematite particles transform to maghemite phase gradually. [20] The ball milling treatment gives a continuous shearing influence on the original α-Fe_2_O_3_ structure, inducing the cationic rearrangement and the adjustment of oxygen planes associated with a lattice expansion. This phenomenon means that the stacking style of iron and oxygen ions tends to adopt the γ-Fe_2_O_3_ structure under a strong shearing stress. On the surface of iron particle under the external force, the stresses are mainly determined by the applied stresses at the very beginning of the surface oxidation process, which is totally different from the free particle surface without the external force. These applied stresses play an important role on the formation of γ-Fe_2_O_3_ nuclei, resulting in γ-Fe_2_O_3_ nanowire growth afterwards. The applied stress assisted growth model of γ-Fe_2_O_3_ nanowires is schematically drawn in Figure 6b. More careful investigation is needed to analyze the initial growth stage of γ-Fe_2_O_3_ nanowires in the future.

## 4. Conclusions

In conclusion, we synthesize γ-Fe_2_O_3_ nanowires by heating iron particles under an external force. TEM and XRD measurements confirm that γ-phase structure appears only under the effect of the applied stresses, whereas α-Fe_2_O_3_ nanowires grow on free iron particles without the external force. The effect of the magnitude of the external force is investigated and a window of the external force is found. An applied stress assisted growth model is proposed to explain the growth of γ-Fe_2_O_3_ nanowires under the external force. We propose that at the very beginning of the surface oxidation process, a strong sheering stress in the surface of the iron oxide layer induces the γ-phase stacking style of iron and oxygen ions, leading to the formation of γ-Fe_2_O_3_ nanowires under the external force.

## References

[1] Chen D., Xiong S., Ran S., Liu B., Wang L., Shen G. (2011). One-dimensional iron oxides nanostructures. Sci. China Phys. Mech. Astron..

[2] Zhu X., Fan J., Zhang Y., Zhu H., Dai B., Yan M., Ren Y. (2017). Preparation of superparamagnetic and flexible γ-Fe_2_O_3_ nanowire arrays in an anodic aluminum oxide template. J. Mater. Sci..

[3] Li Z., Lai X., Wang H., Mao D., Xing C., Wang D. (2009). Direct hydrothermal synthesis of single-crystalline hematite nanorods assisted by 1,2-propanediamine. Nanotechnology.

[4] Chueh Y., Lai M., Liang J., Chou L., Wang Z. (2006). Systematic Study of the Growth of Aligned Arrays of α-Fe_2_O_3_ and Fe_3_O_4_ Nanowires by a Vapor–Solid Process. Adv. Funct. Mater..

[5] Lupan O., Postica V., Wolff N., Polonskyi O., Duppel V., Kaidas V., Lazari E., Ababii N., Faupel F., Kienle L. (2017). Localized Synthesis of Iron Oxide Nanowires and Fabrication of High Performance Nanosensors Based on a Single Fe_2_O_3_ Nanowire. Small.

[6] Liao L., Zheng Z., Yan B., Zhang J., Gong H., Li J., Liu C., Shen Z., Yu T. (2008). Morphology Controllable Synthesis of r-Fe_2_O_3_ 1D Nanostructures: Growth Mechanism and Nanodevice Based on Single Nanowire. J. Phys. Chem. C.

[7] Yuan L., Wang Y., Cai R., Jiang Q., Wang J., Li B., Sharma A., Zhou G. (2012). The origin of hematite nanowire growth during the thermal oxidation of iron. Mater. Sci. Eng. B.

[8] Yuan L., Zhou G. (2012). The Growth of One-Dimensional Oxide Nanostructures by Thermal Oxidation of Metals. Int. J. Nano Sci. Nano Eng. Nanotech..

[9] Birks N., Meier G., Pettit F. (2009). Introduction to High Temperature Oxidation of Metals E.

[10] Rao P., Zheng X. (2011). Unique Magnetic Properties of Single Crystal γ-Fe_2_O_3_ Nanowires Synthesized by Flame Vapor Deposition. Nano Lett..

[11] Wen X., Wang S., Ding Y., Wang Z., Yang S. (2005). Controlled Growth of Large-Area, Uniform, Vertically Aligned Arrays of a-Fe_2_O_3_ Nanobelts and Nanowires. J. Phys. Chem. B.

[12] Cvelbar U., Chen Z., Sunkara M., Mozetic M. (2008). Spontaneous Growth of Superstructure a-Fe_2_O_3_ Nanowire and Nanobelt Arrays in Reactive Oxygen Plasma. Small.

[13] Kim C., Chun H., Kim D., Kim S., Park J., Moon J., Lee G., Yoon J., Jo Y., Jung M. (2006). Magnetic anisotropy of vertically aligned a-Fe_2_O_3_ nanowire array. Appl. Phys. Lett..

[14] Soci C., Bao X., Aplin D., Wang D. (2008). A Systematic Study on the Growth of GaAs Nanowires by Metal-Organic Chemical Vapor Deposition. Nano Lett..

[15] Li B., Yan X., Zhang X., Ren X. (2017). Self-catalyzed Growth of InAs Nanowires on InP Substrate. Nanoscale Res. Lett..

[16] Jensen L., Björk M., Jeppesen S., Persson A., Ohlsson B., Samuelson L. (2004). Role of Surface Diffusion in Chemical Beam Epitaxy of InAs Nanowires. Nano Lett..

[17] Melfo W., Dippenaar R. (2007). In situ observations of early oxide formation in steel under hot-rolling conditions. J. Microsc..

[18] Han Q., Zhang H. (2007). Growth and Properties of Single-Crystalline γ-Fe_2_O_3_ Nanowires. J. Phys. Chem. C.

[19] Shokrollahi H. (2017). A review of the magnetic properties, synthesis methods and applications of maghemite. J. Magn. Magn. Mater..

[20] Randrianantoandro N., Mercier A.M., Hervieu M., Greneche J.M. (2001). Direct phase transformation from hematite to maghemite during high energy ball milling. Mater. Lett..

